# Identification, Efficacy, and Stability Evaluation of Succinimide Modification With a High Abundance in the Framework Region of Golimumab

**DOI:** 10.3389/fchem.2022.826923

**Published:** 2022-04-05

**Authors:** Tao Liu, Jin Xu, Qingcheng Guo, Dapeng Zhang, Jun Li, Weizhu Qian, Huaizu Guo, Xinli Zhou, Sheng Hou

**Affiliations:** ^1^ Department of Oncology, Huashan Hospital, Fudan University, Shanghai, China; ^2^ State Key Laboratory of Antibody Medicine and Targeted Therapy, Shanghai, China; ^3^ NMPA Key Laboratory for Quality Control of Therapeutic Monoclonal Antibodies, Shanghai, China; ^4^ School of Pharmaceutical Sciences, Liaocheng University, Liaocheng, China; ^5^ Shanghai Zhangjiang Biotechnology Co., Ltd., Shanghai, China; ^6^ Taizhou Mabtech Pharmaceuticals Co., Ltd., Taizhou, China

**Keywords:** deamidation, succinimide, golimumab, efficacy, framework region, multi-attribute method

## Abstract

Succinimide (Asu) is the intermediate for asparagine deamidation in therapeutic proteins, and it can be readily hydrolyzed to form aspartate and iso-aspartate residues. Moreover, Asu plays an important role in the protein degradation pathways, asparagine deamidation, and aspartic acid isomerization. Here, Asu modification with a high abundance in the framework region (FR) of golimumab was first reported, the effect of denaturing buffer pH on the Asu modification homeostasis was studied, and the results revealed that it was relatively stable over a pH range of 6.0–7.0 whereas a rapid decrease at pH 8.0. Then, the peptide-based multi-attribute method (MAM) analyses showed that the Asu formation was at Asn 43 in the FR of the heavy chain. Meanwhile, the efficacy [affinity, binding and bioactivity, complement-dependent cytotoxicity (CDC) activity, and antibody-dependent cell-mediated cytotoxicity (ADCC) activity] and stability of the Asu modification of golimumab were evaluated, and the current results demonstrated comparable efficacy and stability between the Asu low- and high-abundance groups. Our findings provide valuable insights into Asu modification and its effect on efficacy and stability, and this study also demonstrates that there is a need to develop a broad-spectrum, rapid, and accurate platform to identify and characterize new peaks in the development of therapeutic proteins, particularly for antibody drugs.

## Introduction

Recombinant therapeutic proteins, mostly produced in Chinese hamster ovary (CHO), murine NS0 cell line, or murine SP2/0 cell line, are becoming common to treat various diseases, such as autoimmune, neoplastic, and infectious diseases (Liu et al., 2014; Ruei-Min Lu et al., 2020; Shanmugaraj et al., 2020; Tambuyzer et al., 2020). To date, approximately 100 Fc-containing protein drugs have been approved by the European Medicines Agency (EMA) and the United States Food and Drug Administration (FDA) for clinical use, and more than 70 are in late-stage development (Reichert, 2012; Ruei-Min Lu et al., 2020; Mullard, 2021; Kaplon et al., 2022).

**FIGURE 1:**
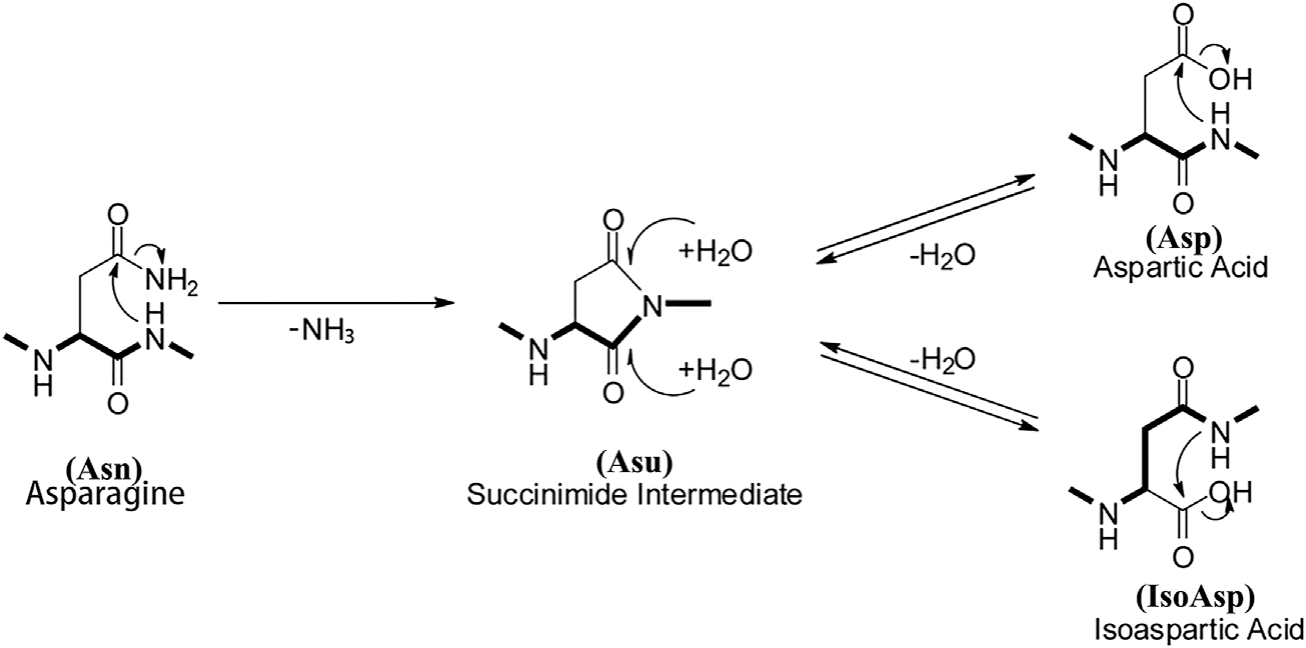
Schematic representation of deamidation pathways.

Therapeutic monoclonal antibodies (mAbs) from different production systems display a much higher degree of heterogeneity due to their posttranslational modifications (PTMs). The PTMs are important product quality attributes that can potentially impact drug safety, efficacy, and stability (Xiaobin Xu et al., 2019). MAbs are subjected to PTMs and degradation during cell culture, purification, storage, and even after administration (Yingda Xu et al., 2019). PTMs, which includes deamidation, isomerization, methionine (Met) and tryptophan (Trp) oxidation, and unpaired cysteine and additional glycosylation in the variable domains, are unique to therapeutic mAbs which could potentially pose higher risk (Liu et al., 2014). The N-terminal pyroGlu formation and the partial removal of C-terminal Lys are two of the well-characterized modifications that cause molecule heterogeneity but have no impact on safety or efficacy. Deamidation, isomerization, and oxidation can compromise the potency, efficacy, and safety of therapeutic antibodies. Glycans attached to therapeutic antibodies might play an important role in their efficacy, pharmacokinetics, and stability (Yingda Xu et al., 2019; Mastrangeli et al., 2020; Vatsa, 2022). One of the most frequent PTMs is the deamidation of asparagine (Asn), a nonenzymatic and spontaneous PTM, which can influence the structure and stability of mAbs (Yang and Zubarev, 2010; Ying and Li, 2022). The deamidation of Asn proceeds through a succinimide (Asu) intermediate. The Asu intermediate can be hydrolyzed to yield Asp or isoAsp (β-Asp), and the isoAsp products are favored at a ratio of about 3:1 (Figure 1) (Ying and Li, 2022).

The effect of deamidation varies depending on the resulting products and the location of the Asn residue. IgG1 Fc deamidation at Asn 325 (EU numbering), which might alter the local structure and interfere with the binding and interaction with the effector cell, is a critical quality attribute for products (Wang et al., 2018; Xiaojun Lu et al., 2020). Several studies have revealed that deamidation in the complementarity determining region (CDR) resulted in decreased activity (Harris et al., 2001; Huang et al., 2005; Vlasak et al., 2009; Yan et al., 2009). As expected, deamidation in the constant domain has no effect on binding affinity (Yan et al., 2009). As a result of deamidation, decreased thermal stability of Fab with isoAsp and increased thermal stability of Fab with Asp compared with the original Fab were observed for a mAb (Vlasak et al., 2009; Liu et al., 2014). The presence of Asu in the CDR regions of several mAbs from Asn deamidation or Asp isomerization resulted in decreased activity (Cacia et al., 1996; Valliere-Douglass et al., 2008; Vlasak et al., 2009; Yan et al., 2009; Yu et al., 2011; Ouellette et al., 2013). However, the impact of Asu in the framework region (FR) was not reported until now. Therefore, the characterization of deamidation-interrelated modifications is important to understand their roles in therapeutic mAbs.

Golimumab, a novel human anti-TNFα IgG1κ mAb, is safe for the treatment of psoriatic arthritis (PsA), rheumatoid arthritis (RA), and ankylosing spondylitis (AS) (Mazumdar and Greenwald, 2009; Kay and Rahman, 2010; Shealy et al., 2010; Melo et al., 2021). Some reports even showed evidence that anti-TNFα treatment enhances the antitumor activity of combined anti-PD-1 and anti-CTLA4 immunotherapy (Haanen et al., 2020). In the present study, golimumab was preliminarily characterized using a subunit-based multi-attribute method (MAM) combined with high-resolution ultra-performance liquid chromatography (UPLC) and ESI–QTOF–MS (LC–MS). An unexpected peak was found in the Fd [the heavy chain (HC) of the Fab] region, which showed a mass shift of -17 Da. We identified and characterized the unexpected peak by the peptide-based MAM. With the traditional sample preparation method (denaturing buffer: 8 M guanidine, 0.1 M Tris/HCl, pH 8.0), there was no unexpected peak with a molecular weight decrease of 17 Da observed. The 17 Da loss modification could potentially be attributed to Asu formation from an aspartic acid or asparagine residue, and the Asu was most likely hydrolyzed during traditional sample preparation (Cao et al., 2019a). Then, the effect of denaturing buffer pH on the modification homeostasis was studied, and the peptide-based MAM analyses confirmed that Asu modification with high abundance in the FR of golimumab was first reported. Meanwhile, the efficacy [affinity, binding and bioactivity, complement-dependent cytotoxicity (CDC) activity, and antibody-dependent cell-mediated cytotoxicity (ADCC) activity] and stability of the Asu modification of golimumab were evaluated, and the results demonstrated comparable efficacy and stability between the Asu low- and high-abundance groups. Our findings provide valuable insights into Asu modification and its effect on efficacy and stability.

## Materials and Methods

### Materials

Golimumab A and golimumab B were manufactured by Shanghai Zhangjiang Biotechnology Co., and purified by Protein A Sepharose (Shanghai Zhangjiang Biotechnology Co.). SIMPONI^®^ (golimumab, Janssen) was obtained from the market of Shanghai, China. Acetonitrile (ACN, MS grade) was obtained from Thermo Scientific (United States); ammonium formate (NH_4_FA), ammonium bicarbonate (NH_4_HCO_3_), sodium cyanotrihydridoborate (NaBH_3_CN), fibrinopeptide (GFP), tris (2-carboxyethyl) phosphine hydrochloride (TCEP), and NaI were all obtained from Sigma (United States). Dithiothreitol (DTT), formic acid (FA, MS grade), and iodoacetamide (MIA) were obtained from Fluka (GER). Trypsin, IdeS (immunoglobulin-degrading enzyme of *Streptococcus pyogenes*), and recombinant human TNFα were obtained from Shanghai Zhangjiang Biotechnology Co. The CHO-K1 and the mouse fibroblastic cell line L929 cell lines were obtained from the American Type Culture Collection (ATCC).

### Instrumentation

Instrumentation separations were performed on the UPLC system (Waters, Milford, MA). All MS measurements were implemented on the Xevo G2-S system (Waters, Milford, MA).

### IdeS Digestion and Reduction

In 2016, we reported the development of a sensitive and high-throughput subunit-based MAM for monitoring the protein fragment (Liu et al., 2016). IdeS of 1 μg was added to each 50 μg of protein. The protein/enzyme mixture was then diluted with 50 mM NH_4_FA, pH 6.6, to a final concentration of 1 mg/ml and incubated at 37°C for 30 min. The IdeS-digested sample was reduced by adding 4 mM TCEP at low pH for 10 min and then analyzed by LC–MS.

### Denaturation of the Samples at Different pH

The samples were diluted to 1 mg/ml, mixed with denaturing buffer (8 M guanidine, 0.1 M Tris/HCl, pH 8.0) or (8 M guanidine, 0.2 M histidine/HCl, pH 6.0, 6.5, 7.0, and 7.5) 20 mM DTT, vortexed, and incubated at 37°C for 1 h, exchanging the buffer to 50 mM NH_4_FA (pH 6.6) and then analyzed by the subunit-based MAM.

### Tryptic Digestion

The denatured samples were buffer exchanged to 50 mM NH_4_FA buffer (pH 6.0) and then digested with trypsin (1:20 w/w) for 4 h at 37°C. The trypsin-digested sample was previously reduced by adding 4 mM TCEP at 37°C for 10 min and then analyzed by the peptide-based MAM.

### Subunit-Based MAM

The UPLC separations of reduced IdeS-digested samples were performed on a BEH300 C4 column (2.1 × 50 mm, 1.7 µm) (Milford, MA, United States). The column was held initially at 80% mobile phase A (0.1% FA in water) for 3 min at 0.40 ml/min, and separation was achieved with a linear gradient from 25 to 35% mobile phase B (0.1% FA in ACN) in 6 min (flow rate: 0.20 ml/min and column temperature: 60°C). Typically, 0.5 μg of the sample was injected for MS analysis with positive ion mode on a Xevo G2-S Q-TOF. The m/z scan range was 500–2,500. The desolvation gas and cone gas flows were 800 and 50 L/h, respectively.

### Peptide-Based MAM

The trypsin-digested samples were centrifuged at 17,000 g for 10 min, and the supernatant was analyzed by LC–MS/MS on a BEH300 C18 column (2.1 × 100 mm, 1.7 µm) (Waters, Milford, MA) with a column temperature of 45°C and 80-min linear gradient (1–37% phase B) at 0.20 ml/min. The column was equilibrated with 1% B in water. The LC mobile phases were the same as the previous subunit-based MAM. Data were obtained with positive ionization. Eluted peptides were detected by MS with an alternating low-collision energy (6 V) and high-collision energy (ramping from 25 to 45 V) ESI + acquisition mode. Scan time was 0.5 s. The ion source setup was presented as follows: capillary voltage, 3,000 V; the desolvation temperature, 350°C; source temperature, 120°C; cone voltage, 40 V; cone gas flow, 50 L/h; the desolvation gas flow, 800 L/h; and scan range, 50–2500. The data analysis was performed with mass accuracy no more than 30 ppm.

### Binding Assay

ELISA was used to determine the binding activity of golimumab. Recombinant human TNFα was used to capture samples. Goat anti-human IgG conjugated to horseradish peroxidase (HRP) was then added to detect bound golimumab. Subsequent to washing, tetramethylbenzidine was added. Prior to absorbance readings at 450 nm, reaction quenching was achieved *via* acidification with sulfuric acid. A four-parameter fit model was used to determine BC50 values. The binging activity of golimumab was determined by comparing its ability to be captured by TNFα relative to the reference standard (SIMPONI^®^).

### L929 Potency Assay

This assay was performed according to the previous report (Tebbey et al., 2015). The potency of golimumab was tested in the L929 cell line using human TNFα. Before being added to the L929 cells, various concentrations of golimumab were mixed with a constant amount of TNFα. Cell viability was measured using the tetrazolium dye (MTT). The potency of golimumab was determined by comparing its neutralizing effect relative to the reference standard (SIMPONI^®^).

### CDC Bioassay

The CDC activities of golimumab were evaluated by a complement-mediated cell-killing assay. Briefly, 1 × 10^5^ CHO-K1/hmTNFα cells were incubated with 6.7% (v/v) complement (Cedarlane Labs, Burlington, Canada) and samples for 1 h. The lactate dehydrogenase activity was measured by a Cytotoxicity Detection Kit (Roche Diagnostics, Indianapolis, IN). The CDC activity was expressed as percent cytotoxicity relative to that lysed by 0.67% (v/v) Triton X-100.

### ADCC-Reporter Gene Bioassay

This assay was performed according to the previous report (Yang et al., 2015). CHO-K1/hmTNFα cells (overexpressed TNFα) were seeded at 10,000 cells per well in a 96-well opaque tissue culture plate. Golimumab was serially diluted and incubated with the CHO-K1/hmTNFα cells for approximately 1 h at 37°C, 5% CO_2_. Following incubation, Jurkat-expressing NFAT-Re/CD16a (158 V)-FcRγ/pcDNA3.1 (hygro) luciferase reporter cells (engineered Jurkat cells stably expressing the human FcγRIIIa receptor, V158 variants, and an NFAT response element driving the expression of luciferase) were added to the CHO-K1/hmTNFα/antibody mixture. The mixture was incubated for approximately 4 h at 37°C, 5% CO_2_, and then measured for luciferase production using a luminescent substrate (Promega Bright Glo).

### SPR Assay

The kinetics and affinity of golimumab were determined by SPR using a Biacore T200 (GE Healthcare). Anti-human Fc antibody (GE Healthcare), diluted to 20 mg/ml in buffer (10 mM sodium acetate, pH 5.0), was immobilized on a CM5 chip (GE Healthcare). Test antibodies of 2.6 mg/ml was captured on the chip, and rate equations derived from the 1:1 Langmuir binding model were fitted for kinetic analysis with the use of T200 evaluation software, version 1.0. Kinetic rate constants for the association (K_a_, 1/Ms) and dissociation rate constants (K_d_, 1/s) were determined under the flow rate of 30 μL/min at 25°C. The rate constants were derived by making kinetic binding measurements at five different antigen concentrations (7.81–125 nM). The equilibrium dissociation rate constant (K_D_, M) was calculated by the formula: K_D_ = K_d_/K_a_.

### The Accelerated Stability Studies

Accelerated stability and forced degradation studies were performed according to previous reports (Bardo-Brouard et al., 2016; Tamizi and Jouyban, 2016; Nowak et al., 2017a). Stability studies were performed accelerated at 25°C for 6 months and stress temperature 40°C for 10 days. The stability samples were taken at different time points and analyzed using capillary electrophoresis-sodium dodecyl sulfate (CE-SDS), size-exclusion chromatography (SEC), and ion-exchange chromatography (IEX). Binding and bioactivity were also performed as previously described.

### Statistical Analysis

Data are expressed as mean ± SD or mean ± SEM. Statistical analysis was conducted by Student’s two-sided unpaired *t*-test unless otherwise indicated. Differences were considered significant at *p* < 0.05 (*). All statistic calculations were performed using GraphPad Prism 9 software.

## Results

### Mass Spectral Analsysis of Reduced IdeS Digests by Subunit-Based MAM

**FIGURE 2:**
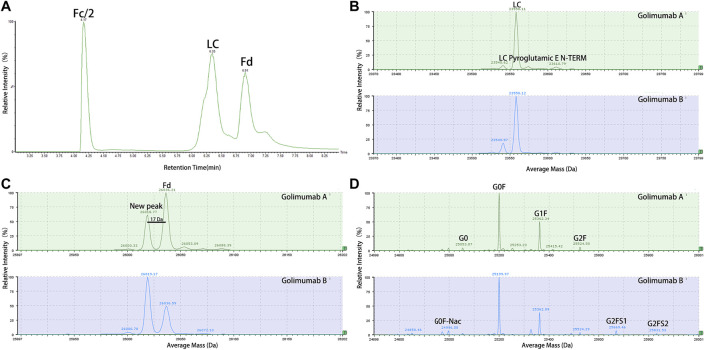
Unexpected peak was detected in the Fd region of golimumab by the subunit-based MAM. Golimumab was digested by IdeS and reduced by TCEP and then analyzed by the subunit-based MAM (TIC). **(A)** Deconvoluted mass spectrum of the LC **(B)**, Fd **(C)**, and Fc/2 **(D)** of golimumab A and B.

**FIGURE 3:**
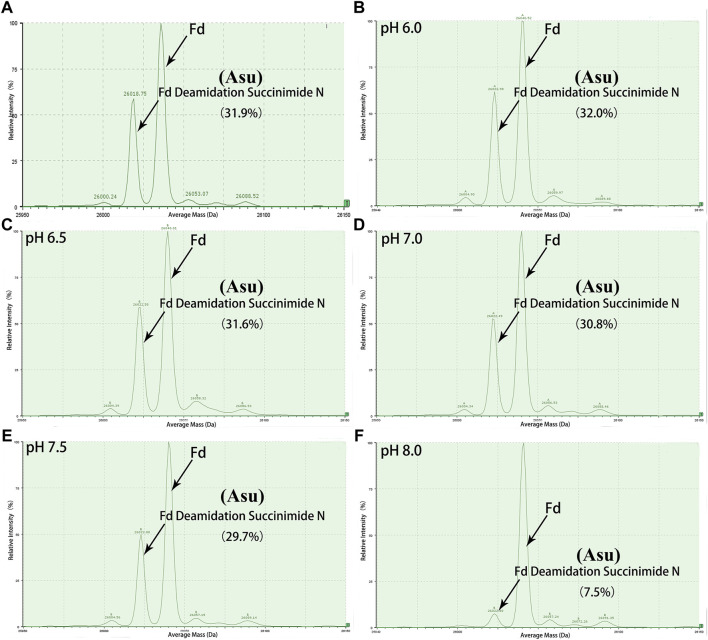
Deconvoluted mass spectrum of golimumab Fd treated with buffers of different pH. An unexpected peak in Fd was relatively stable in the pH range of 6.0–7.0 and decreased rapidly as pH increased from 7.5 to 8.0. **(A)** control, **(B)** denaturation of the sample at pH 6.0, **(C)** denaturation of the sample at pH 6.5, **(D)** denaturation of the sample at pH 7.0, **(E)** denaturation of the sample at pH 7.5, **(F)** denaturation of the sample at pH 8.0.

The MAM was developed to monitor and quantify multiple PTMs of biotherapeutics (Rogstad et al., 2019). In this study, golimumab was digested by IdeS and separated into Fc/2, Fd, and LC after reduction. A subunit-based MAM method which combined UPLC and MS was used to measure the molecular masses of Fc/2, Fd, and LC (Figure 2A). An unexpected peak was found in the Fd region, which showed a mass shift of −17 Da. For further study of the unexpected peak, we produced two golimumab variants (golimumab A and B) with low and high abundance of the unexpected peak, and the relative amounts of unexpected peak were approximately 31.9 and 60.4% in golimumab A and B (about 55–60% in SIMPONI^®^, data not shown), respectively (Figure 2B and Figure 3A). Other modification (pyroglutamic acid E N-TERM and N-glycosylation and N-lysine C-TERM) patterns of golimumab A and B were remarkably similar, which are shown in Figures 2B–D.

### Identification and Characterization of Asu Modification by Peptide-Based MAM

**FIGURE 4:**
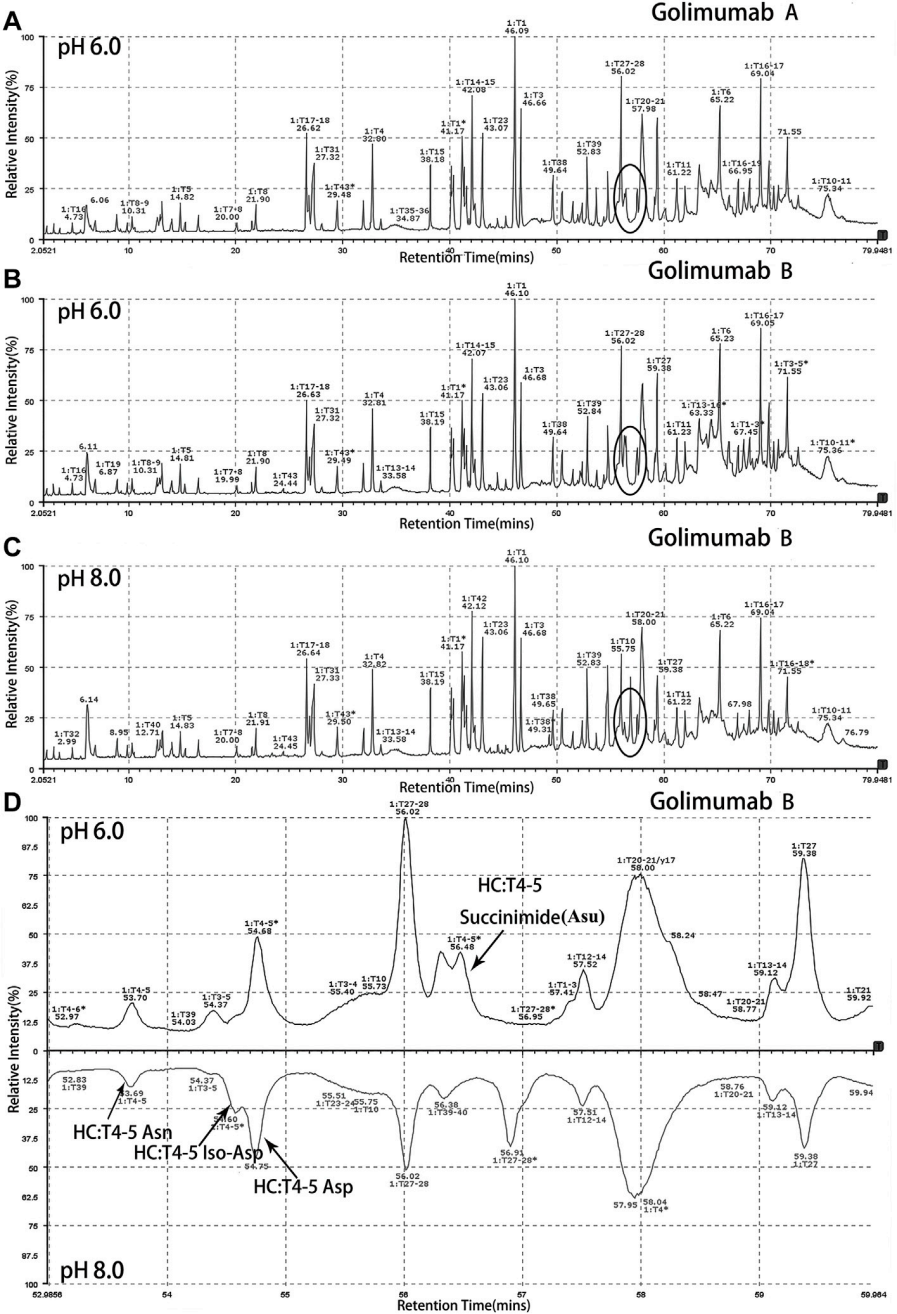
Identification and characterization of the unexpected peak by the peptide-based MAM. An unexpected peak eluting at about 56.5 min was detected [**(A)**: golimumab A pH 6.0, **(B)**: golimumab B pH 6.0, **(C)**: golimumab B pH 8.0, and **(D)**: comparison of golimuamb B at pH 6.0 and pH 8.0].

**FIGURE 5:**
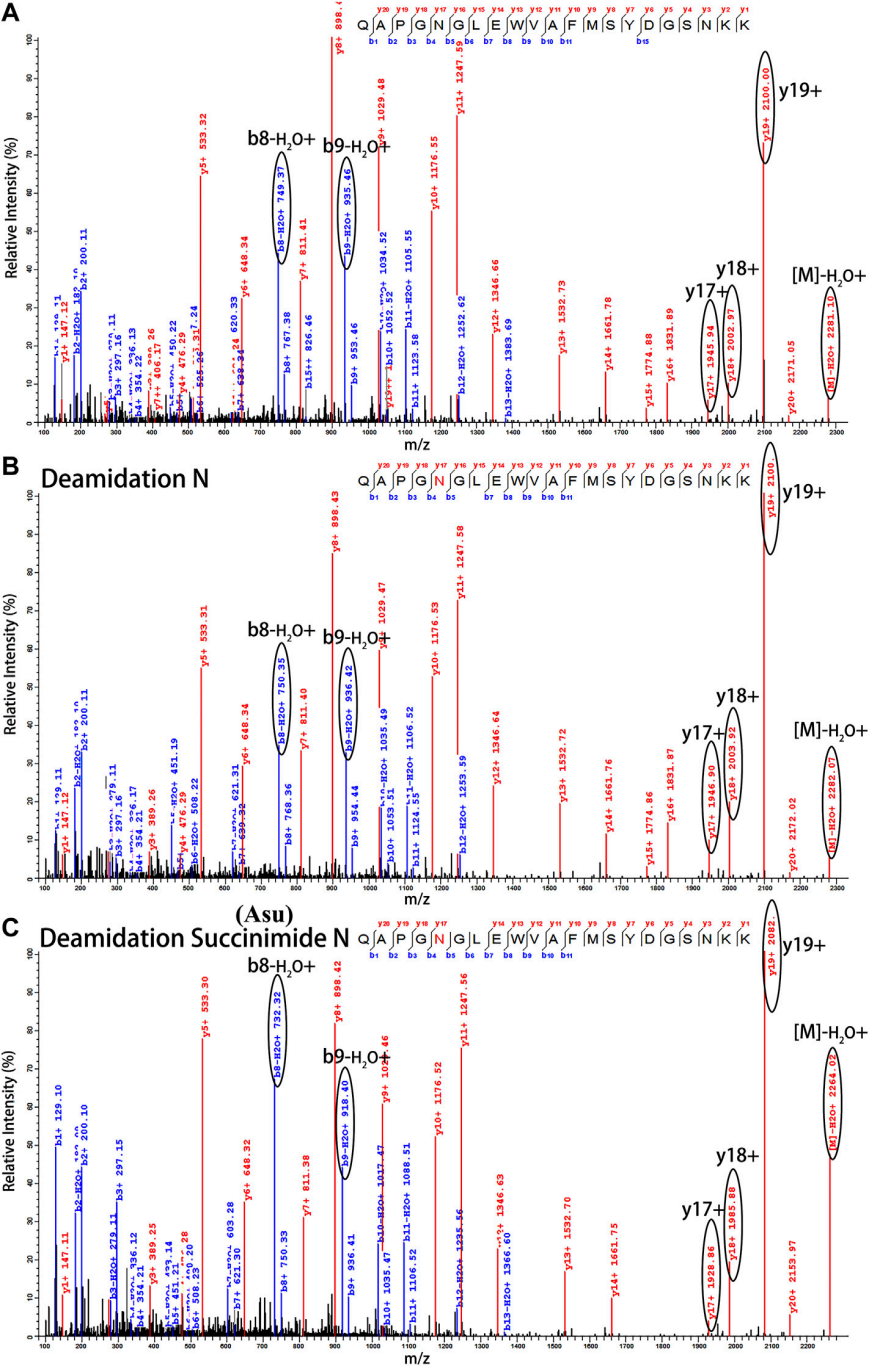
Identification of Asu modification in the FR of golimumab. MS/MS spectra of precursor ions at **(A)** m/z of 767.0142 for native peptide HC: H4-5 (QAPGNGLEWVAFMSYDGSNKK) and **(B)** m/z of 767.3643 for deamidation and **(C)** m/z of 761.3448 for Asu. Fine mapping and analysis by a mass spectral peak-labeling software (pLabel^™^) (Li et al., 2005; Wang et al., 2007).

**FIGURE 6:**
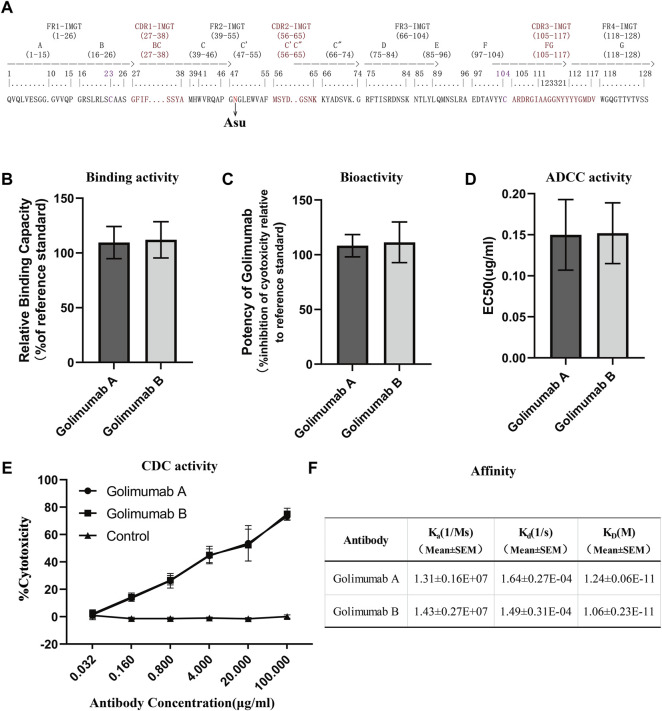
Efficacy evaluation of Asu modification with a high abundance in golimumab. **(A)** Three heavy-chain CDRs, FRs, and Asu modification site of golimumab. Binding **(B)** and bioactivity **(C)**, ADCC activity **(D)**, and CDC **(E)** activity and affinity **(F)** evaluation of golimumab A and golimumab B.

Peptide-based MAM was one of the broad-spectrum, rapid, and accurate methods for unexpected peak detection (NPD), and MAM offers the advantage of measuring multiple protein modifications as product quality attributes during development or critical quality attributes (CQAs) during testing in a single-MS run (Rogstad et al., 2019). In this study, we identified and characterized the unexpected peak by the peptide-based MAM. With the traditional sample preparation method (denaturing buffer: 8 M guanidine, 0.1 M tris/HCl, pH 8.0), there was no unexpected peak with a molecular weight decrease of 17 Da observed in golimumab B (Figure 4C). Therefore, it was necessary to determine whether the denaturing buffer affected the detection of the unexpected peak. First, the effect of the buffer pH on the unknown modification homeostasis in Fd was studied. The samples previously digested by IdeS were treated with denaturing buffers of pH 6.0, 6.5, 7.0, 7.5, and 8.0 and then analyzed by the subunit-based MAM. As seen in the deconvoluted MS spectrum of Fd (golimumab A) shown in Figure 3, the relative abundance of the unexpected peak decreased from 32.0 to 7.5% with the increase of pH from 6.0 to 8.0. On the other hand, the relative abundance of the unexpected peak was relatively stable in the pH range of 6.0–7.0 and decreased rapidly as pH increased from 7.5 to 8.0. The 17 Da loss modification could potentially be attributed to Asu formation from an aspartic acid or asparagine residue, and the Asu was most likely hydrolyzed during traditional sample preparation. In order to further identify and characterize the modification, golimumab A and B were denatured in 0.2 M His–HCl and 8 M Gua–HCl, pH 6.0, and digested in 50 mM NH_4_FA, pH 6.0, and then analyzed by the peptide-based MAM. Compared with the traditional sample preparation method (Figure 4C), an unexpected peak eluting at about 56.5 min was detected (Figures 4A, B, D). The extracted mass spectrum showed the molecular ion peak at m/z 761.3448. The mass of the unexpected peak is 2281.0437 Da, and it did not match the expected protein sequence according to the data analysis. The molecular weight (MW) was 17 Da lighter than the T4-5 tryptic peptide of the HC (QAPGN*GLEWVAFMSYDGSNKK, m/z 767.0142), and this result is in consistence with that of the subunit-based MAM. The MS/MS spectra analysis with collision-induced dissociation (CID) of the two peptides (golimumab B, pH 6.0/8.0) is shown in Figures 5A, C. Compared with the MS/MS spectra of HC T4-5 tryptic peptide, the unexpected peptide demonstrated no mass shift in y16 ions, and a mass shift of -17Da is shown in y17–y20 ion (Figure 5C). The hypothesis of Asu was proposed as a result of the decreased mass of −17 Da. Moreover, at low pH, Asu is stable and can be analyzed with accurate mass measurements and tandem MS to confirm its identity and localize its modification site. Conversely, under neutral to basic conditions, Asu hydrolysis occurs more rapidly than Asu formation, resulting in the formation of Asp and isoAsp and a low amount of Asu remaining (Cao et al., 2019a; Cao et al., 2019b). In further analysis, the deamidation of HC T4-5 (at 54.60 and 54.75 min) was identified by MS/MS (Figure 5B). Therefore, it is reasonable to believe that the unexpected peptide is Asu formation at Asn 43 of HC T4-5 (Figure 6A).

Along with this, the proportions of deamidation and Asu were calculated in each site at different pH conditions (pH 6.0 and 8.0). The results are shown in Table 1. The percentage of Asu in golimumab A at the peptide level was nearly consistent (28.2 and 31.9%) with that at the subunit level. Using the peptide-level analysis of MAM, the relative abundance of Asu in golimumab B decreased significantly from 54.4 to 4.1% with the increase of pH from 6.0 to 8.0. Meanwhile, the proportions of deamidation in golimumab B were found increased dramatically in HC T4-5, T27-28, and T38 tryptic peptide at pH 8.0, which were 73.4, 43.0, and 16.2%, respectively. They were all further confirmed by MS/MS (Supplementary Figure S1 and S2).

**TABLE 1:** Analysis of deamidation and its interrelated modifications in golimumab denatured at pH 6.0 and 8.0 by peptide-based MAM.

Peptide and modification	Sequence	Theoretical mass	Observed mass	Golimumab A (pH 6.0)	Golimumab B (pH 6.0)	Golimumab B (pH 8.0)
HC:T4-5	QAPGN*GLEWVAFMSYDGSNKK	2298.0740	2298.0801	52.0 ± 1.6%	22.4 ± 0.5%	22.6 ± 4.5%
HC:T4-5 deamidation		2299.0579	2299.0579	19.8 ± 3.7%	23.3 ± 1.6%	73.4 ± 6.0%
HC:T4-5 Asu		2281.0474	2281.0437	28.2 ± 2.0%	54.4 ± 1.1%	4.1 ± 1.5%
HC:T27-28	VVSVLTVLHQDWLN*GKEYK	2227.2000	2227.2268	99.2 ± 0.3%	96.6 ± 1.4%	54.1 ± 4.9%
HC:T27-28 deamidation		2228.1841	2228.1816	0.1 ± 0.1%	1.7 ± 0.9%	43.0 ± 6.3%
HC:T27-28 Asu		2210.1736	2210.1745	0.7 ± 0.2%	1.7 ± 0.5%	2.9 ± 1.4%
HC:T38	GFYPSDIAVEWESN*GQPENNYK	2543.1240	2543.1221	99.5 ± 0.1%	99.2 ± 0.2%	82.3 ± 0.8%
HC:T38 deamidation		2544.1082	2544.1313	0.0 ± 0.0%	0.1 ± 0.2%	16.2 ± 1.3%
HC:T38 Asu		2526.0977	2526.1143	0.5 ± 0.1%	0.7 ± 0.0%	1.5 ± 0.5%

“*” indicates the modification site.

### Efficacy Evaluation of Asu Modification in Golimumab

To some extent, though, it might still be challenging to fully understand the effect of Asu modification. Under all circumstances, it was important to assess the PTM with regard to efficacy, safety, and stability. Therefore, information on Asu modification is critical to establish the relationship between the quality attribute and efficacy, with the ultimate goal of ensuring the efficacy and safety of therapeutic monoclonal antibodies.

### Affinity

Affinity for the soluble human tumor necrosis factor (TNFα) trimer was determined by surface plasmon resonance (SPR) using a Biacore T200 (Cytiva). For binding of human TNFα, anti-human IgG antibody was covalently immobilized on a CM5 chip (Biacore AB), and the kinetic binding parameters as well as the affinity constants for the binding between TNFα and golimumab A/B were determined by Biacore analysis and compared with the binding parameters for the interaction between TNFα and golimumab. SPR measurements indicated that there was no noticeable difference between golimumab A (K_D_ = 1.24 ± 0.06E-11M) and B (K_D_ = 1.06 ± 0.23E-11M) in Ka, Kd, and KD (Figure 6F). The results showed that Asu modification at Asn 43 had no significant effect on the affinity of golimumab to TNFα.

### Binding and Bioactivity

A competitive binding assay was then conducted to further verify the binding activity of golimumab A and B. Moreover, the inhibitory effect of golimumab on TNFα activity was tested with L929 cells for TNFα-induced cell death. It was shown that the relative binding and bioactivity of golimumab A were similar to those of golimumab B (Figures 6B, C). These results suggested that the Asu modification at Asn 43 had almost no effect on binding and bioactivity of golimumab.

### ADCC and CDC Activities

Previous studies have demonstrated that afucosylated Fc-region carbohydrates of antibody increased its affinity for FcγRIIIa and enhanced ADCC activity (Chung et al., 2012; Yang et al., 2015) and a positive correlation between the galactose content and CDC activity (Hodoniczky et al., 2005), due to the overriding effects of the afucosylated glycoforms and galactosylation, the impact of other sugar residues on ADCC or CDC activity is less clear (Xie et al., 2020). Accordingly, we investigated the glycosylation pattern of golimumab. The glycosylation patterns of golimumab A and B were remarkably similar, which is shown in Figure 2D. Ruling out the impact of glycosylation on ADCC and CDC activities, the ADCC reporter gene assay was then used for evaluating the ADCC activity (Parekh et al., 2012). The Jurkat cell line that stably expresses the FcγRIIIa complex and the luciferase reporter gene under the control of the NFAT response elements were chosen as the effector cells. CHO-K1/hmTNFα cells, which overexpressed TNFα, were used as target cells. ADCC activity, which is largely dependent on the interaction of golimumab and FcγR on macrophages and NK cells, was highly similar between golimumab A and B; the EC50 were approximately 0.150 μg/ml and 0.152 μg/ml, respectively (Figure 6D).

The CDC activity was determined by complement-mediated cell-killing assay. CDC assay showed golimumab B was capable of killing cells in a markedly dose-dependent manner in the CHO-K1/hmTNFα cell line. In addition to ADCC activity, triggering of CDC mediated by complement binding of golimumab was also similar between golimumab A and B (Figure 6E). These findings suggested that the Asu modification of golimumab had little effect on CDC and ADCC activities.

### Stability Evaluation of Asu Modification in Golimumab

**FIGURE 7:**
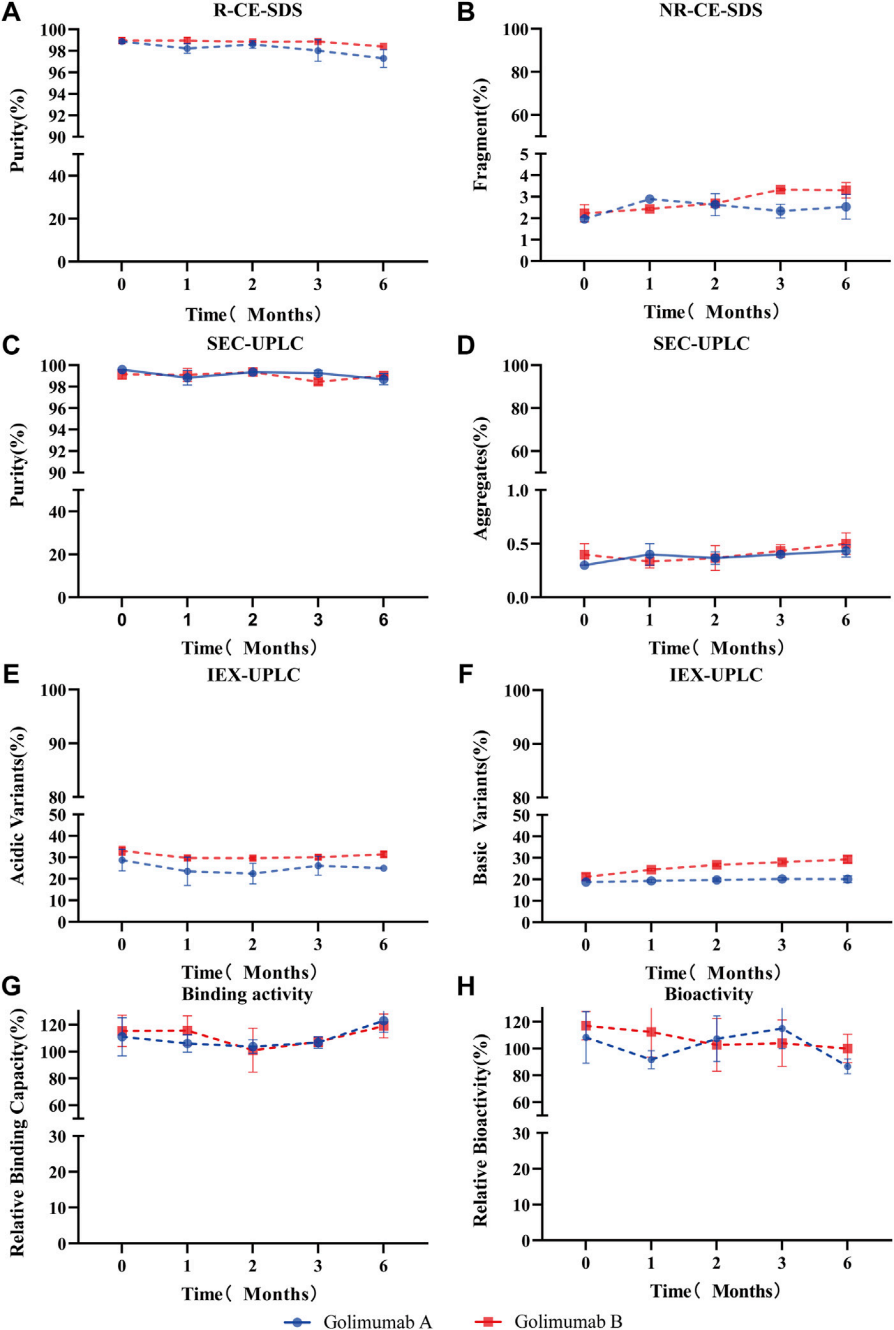
Accelerated stability evaluation of Asu modification with high abundance in golimumab. Accelerated stability samples were analyzed using R (reduced)-CE-SDS **(A)**, NR (non-reduced)-CE-SDS **(B)**, SEC-UPLC [purity: **(C)**, aggregates **(D)**], IEX [acidic variants **(E)**, basic variants **(F)**], binding activity **(G)**, and bioactivity **(H)** at 25°C at the (0, 1, 2, 3, and 6) month time points.

For long-term storage, deamidation of mAbs produces acidic variants, which may alter the pharmacokinetics and antigenicity, and thus affects the efficacy and shelf life of mAb therapeutics. Therefore, the asparagine deamidation and aspartic acid isomerization may affect the long-term stability and biological activity of mAbs. So, the accelerated stability and forced degradation studies were performed to assess the stability of the Asu modification in golimumab. Similar stability profiles and degradation trends (the purity, fragments, aggregates, acidic variants, basic variants, binding, and bioactivity) under accelerated/stress conditions were observed (Figure 7; Supplementary Figure S3). The results demonstrated comparable stability between the Asu low- and high-abundance groups; the Asu intermediate modification at Asn 43 in the variable region of golimumab has virtually no effect on stability.

## Discussion

TNFα plays a key role in the pathogenesis of a variety of diseases. The reduction of TNFα levels has been shown to ameliorate symptoms of diseases, including RA, PsA, and AS (Gottlieb, 2007). Drugs targeting TNFα have greatly improved prognosis for patients with RA over the past decades and slowed the progression of joint damage. Golimumab, a novel human anti-TNFα IgG1κ monoclonal antibody with complex modifications, was approved in April 2009 by FDA as a treatment for three separate conditions.

Deamidation, a spontaneous nonenzymatic reaction, is one of the most important PTMs. Some previous studies have identified that deamidation in proteins can impact stability and potency (Ouellette et al., 2013; Zhang et al., 2014; Lu et al., 2019; Xiaojun Lu et al., 2020). Usually, this reaction is associated with protein degradation, triggering apoptosis in cancer cells as well as other regulatory functions. Under physiological conditions, it can format a five-membered Asu ring intermediate by the nucleophilic attack of the nitrogen atom in the following peptide bond on the carbonyl group of the Asn side chain (Yang and Zubarev, 2010; Ying and Li, 2022). Due to the great diversity of potential deamidation positions and its interrelated modifications (Asp/IsoAsp/Asu), the influence of deamidation is likely to be variable. Therefore, developing a broad-spectrum, rapid, and accurate platform to evaluate deamidation-interrelated modifications or other PTMs is still needed. As a representative method of broad-spectrum, rapid, and accurate platform, MAMs were developed to monitor and quantify multiple PTMs of biotherapeutics (Rogstad et al., 2019). In this study, a subunit-based MAM and peptide-based MAM were used to characterize or control the PTMs of golimumab, and an Asu intermediate with a high abundance was first identified and evaluated in the FR of the heavy chain.

**FIGURE 8:**
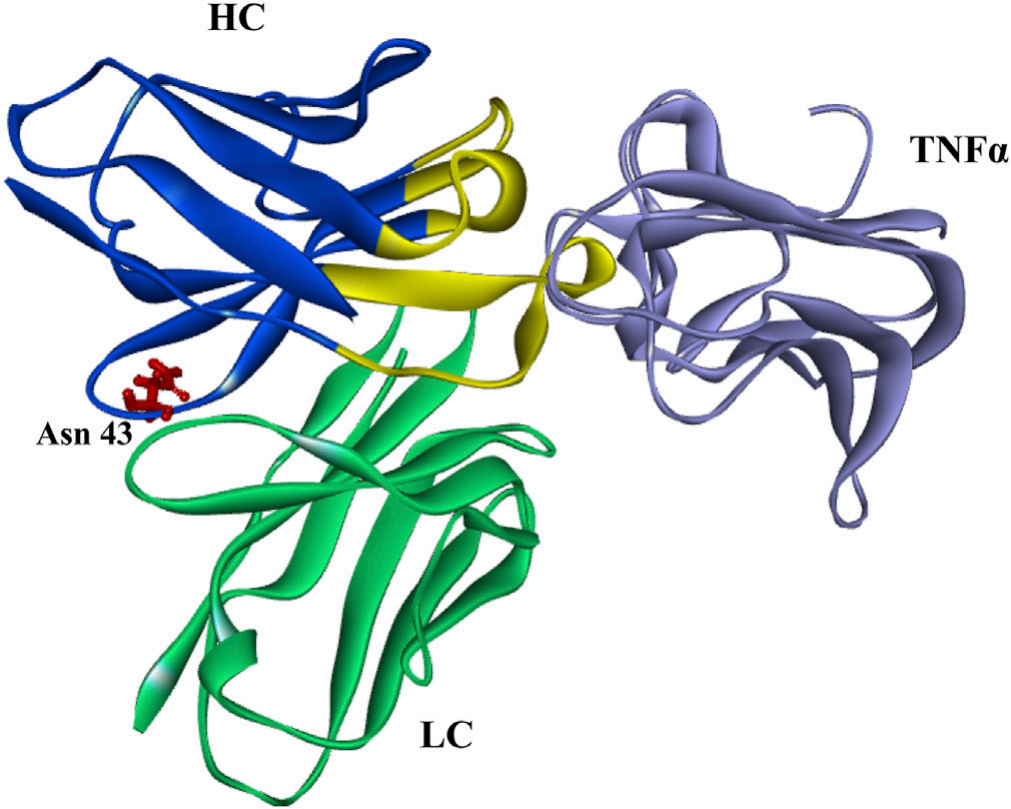
Overall structure of the TNFa–golimumab complex (FRs: blue and CDRs: yellow) (Ono et al., 2018).

The rate of deamidation is affected by the protein structure (from primary to higher-order structure), temperature, pH, and other environmental factors (Scotchler and Robinson, 1974; Pace et al., 2013). The effects of pH are relatively complex that deamidation may be accelerated under neutral or alkaline conditions, while Asu is relatively stable under acidic ones (McCudden and Kraus, 2006; Ouellette et al., 2013; Nowak et al., 2017b; Cao et al., 2019a; Lu et al., 2019). While pH is important for Asu formation, other factors such as primary/higher-order structure, temperature, buffer composition, and ionic strength are reported to play significant roles (Ouellette et al., 2013). In present study, the effect of denaturing buffer pH on Asu modification homeostasis was studied. The Asu formation in FR was relatively stable over a pH range of 6.0–7.0 whereas decreased rapidly at pH 8.0. We also found that the Asu formation in the FR of the heavy chain was relatively stable with a slight decreasing tendency when samples were denatured without DTT at different pH (data not shown). We suspected it might have some connection with the antibody structure, and the Asu modification at Asn 43 was within the FR not CDRs which emerges from the surface of golimumab to form the surface that recognizes TNFα. This was further supported by the overall structure of the TNFa–golimumab complex shown in Figure 8 (Ono et al., 2018). With the traditional peptide mapping method (denaturing buffer: 8 M guanidine, 0.1 M Tris/HCl, pH 8.0), the proportions of deamidation were found increased dramatically in each site. Further investigation revealed that the deamidation rate was dependent on the buffer pH.

As the great diversity of potential modification sites, the influence of deamidation and its interrelated modifications is likely to be variable. Deamidation at Asn 325 of IgG1 Fc is a critical quality attribute for products whose mechanism of action includes ADCC (Xiaojun Lu et al., 2020). Deamidation in the CDRs resulted in decreased binding affinity and potency (Harris et al., 2001; Huang et al., 2005; Vlasak et al., 2009; Yan et al., 2009). The presence of Asu in the CDRs of several antibodies from Asn deamidation or Asp isomerization resulted in decreased binding and bioactivity (Cacia et al., 1996; Valliere-Douglass et al., 2008; Vlasak et al., 2009; Yan et al., 2009; Yu et al., 2011; Ouellette et al., 2013). However, the impact of Asu with a high abundance in the FR has not reported until now. In this study, the efficacy (affinity, binding and bioactivity, and CDC and ADCC activities) of Asu modification at Asn 43 in FR of golimumab was evaluated. The result indicated that the Asu intermediate modification at Asn 43 in the variable region of golimumab has virtually no effect on the efficacy (affinity, binding and bioactivity, and CDC and ADCC activities) and stability. It may be that the Asu modification at Asn 43 was within the FR not CDRs, which emerge from the surface of golimumab to form the surface that recognizes TNFα. It is not inconsistent with previous reports.

## Conclusion

To the best of our knowledge, this study is the first detailed analysis of the Asn deamidation Asu intermediate modification with a high abundance in the FR of golimumab using a subunit-based MAM and peptide-based MAM. We first identified the modification and evaluated the efficacy and stability. The results indicated that the Asu modification at Asn 43 in the FR of golimumab has virtually no effect on the efficacy and stability. Our findings provide valuable insights into Asu modification and its effect on efficacy and stability; this study also demonstrates that there is a need to develop a broad-spectrum, rapid, and accurate platform to identify and characterize new peaks in the development of therapeutic proteins, particularly for antibody drugs. Due to the importance of deamidation and its interrelated modifications, further studies need to be carried out.

## Data Availability

The original contributions presented in the study are included in the article/Supplementary Material, and further inquiries can be directed to the corresponding authors.

## References

[B1] Bardo-Brouard P. Vieillard V. Shekarian T. Marabelle A. Astier A. Paul M. (2016). Stability of Ipilimumab in its Original Vial after Opening Allows its Use for at Least 4 Weeks and Facilitates Pooling of Residues. Eur. J. Cancer 58, 8–16. 10.1016/j.ejca.2016.01.008 26922168

[B2] Cacia J. Keck R. Presta L. G. Frenz J. (1996). Isomerization of an Aspartic Acid Residue in the Complementarity-Determining Regions of a Recombinant Antibody to Human IgE: Identification and Effect on Binding Affinity. Biochemistry 35, 1897–1903. 10.1021/bi951526c 8639672

[B3] Cao M. Mulagapati S. H. R. Vemulapalli B. Wang J. Saveliev S. V. Urh M. (2019a). Characterization and Quantification of Succinimide Using Peptide Mapping under Low-pH Conditions and Hydrophobic Interaction Chromatography. Anal. Biochem. 566, 151–159. 10.1016/j.ab.2018.11.021 30503708

[B4] Cao M. Xu W. Niu B. Kabundi I. Luo H. Prophet M. (2019b). An Automated and Qualified Platform Method for Site-specific Succinimide and Deamidation Quantitation Using Low-pH Peptide Mapping. J. Pharm. Sci. 108, 3540–3549. 10.1016/j.xphs.2019.07.019 31374319

[B5] Chung S. Quarmby V. Gao X. Ying Y. Lin L. Reed C. (2012). Quantitative Evaluation of Fucose Reducing Effects in a Humanized Antibody on Fcγ Receptor Binding and Antibody-Dependent Cell-Mediated Cytotoxicity Activities. MAbs 4, 326–340. 10.4161/mabs.19941 22531441 PMC3355491

[B6] Gottlieb A. B. (2007). Tumor Necrosis Factor Blockade: Mechanism of Action. J. Invest. Dermatol. Symp. Proc. 12, 1–4. 10.1038/sj.jidsymp.5650029 17502861

[B7] Haanen J. Ernstoff M. S. Wang Y. Menzies A. M. Puzanov I. Grivas P. (2020). Autoimmune Diseases and Immune-Checkpoint Inhibitors for Cancer Therapy: Review of the Literature and Personalized Risk-Based Prevention Strategy. Ann. Oncol. 31, 724–744. 10.1016/j.annonc.2020.03.285 32194150

[B8] Harris R. J. Kabakoff B. Macchi F. D. Shen F. J. Kwong M. Andya J. D. (2001). Identification of Multiple Sources of Charge Heterogeneity in a Recombinant Antibody. J. Chromatogr. B: Biomed. Sci. Appl. 752, 233–245. 10.1016/s0378-4347(00)00548-x 11270864

[B9] Hodoniczky J. Zheng Y. Z. James D. C. (2005). Control of Recombinant Monoclonal Antibody Effector Functions by Fc N-Glycan Remodeling *In Vitro* . Biotechnol. Prog. 21, 1644–1652. 10.1021/bp050228w 16321047

[B10] Huang L. Lu J. Wroblewski V. J. Beals J. M. Riggin R. M. (2005). *In Vivo* deamidation Characterization of Monoclonal Antibody by LC/MS/MS. Anal. Chem. 77, 1432–1439. 10.1021/ac0494174 15732928

[B11] Kaplon H. Chenoweth A. Crescioli S. Reichert J. M. (2022). Antibodies to Watch in 2022. MAbs 14, 2014296. 10.1080/19420862.2021.2014296 35030985 PMC8765076

[B12] Kay J. Rahman M. U. (2010). Golimumab: A Novel Human Anti-TNF-alpha Monoclonal Antibody for the Treatment of Rheumatoid Arthritis, Ankylosing Spondylitis, and Psoriatic Arthritis. Core Evid. 4, 159–170. 10.2147/ce.s6000 20694072 PMC2899784

[B13] Li D. Fu Y. Sun R. Ling C. X. Wei Y. Zhou H. (2005). pFind: a Novel Database-Searching Software System for Automated Peptide and Protein Identification via Tandem Mass Spectrometry. Bioinformatics 21, 3049–3050. 10.1093/bioinformatics/bti439 15817687

[B14] Liu H. Ponniah G. Zhang H.-M. Nowak C. Neill A. Gonzalez-Lopez N. (2014). *In Vitro* and *In Vivo* Modifications of Recombinant and Human IgG Antibodies. mAbs 6, 1145–1154. 10.4161/mabs.29883 25517300 PMC4622420

[B15] Liu T. Guo H. Zhu L. Zheng Y. Xu J. Guo Q. (2016). Fast Characterization of Fc-Containing Proteins by Middle-Down Mass Spectrometry Following IdeS Digestion. Chromatographia 79, 1491–1505. 10.1007/s10337-016-3173-2

[B16] Lu X. Nobrega R. P. Lynaugh H. Jain T. Barlow K. Boland T. (2019). Deamidation and Isomerization Liability Analysis of 131 Clinical-Stage Antibodies. mAbs 11, 45–57. 10.1080/19420862.2018.1548233 30526254 PMC6343770

[B17] Mastrangeli R. Audino M. C. Palinsky W. Broly H. Bierau H. (2020). The Formidable Challenge of Controlling High Mannose-Type N-Glycans in Therapeutic mAbs. Trends Biotechnol. 38, 1154–1168. 10.1016/j.tibtech.2020.05.009 32616303

[B18] Mazumdar S. Greenwald D. (2009). Golimumab. mAbs 1, 422–431. 10.4161/mabs.1.5.9286 20065639 PMC2759491

[B19] McCudden C. R. Kraus V. B. (2006). Biochemistry of Amino Acid Racemization and Clinical Application to Musculoskeletal Disease. Clin. Biochem. 39, 1112–1130. 10.1016/j.clinbiochem.2006.07.009 17046734

[B20] Melo A. T. Campanilho-Marques R. Fonseca J. E. (2021). Golimumab (Anti-TNF Monoclonal Antibody): where We Stand Today. Hum. Vaccin. Immunother. 17, 1586–1598. 10.1080/21645515.2020.1836919 33369527 PMC8115761

[B21] Mullard A. (2021). FDA Approves 100th Monoclonal Antibody Product. Nat. Rev. Drug Discov. 20, 491–495. 10.1038/d41573-021-00079-7 33953368

[B22] Nowak C. K. Cheung J. M. Dellatore S. Katiyar A. Bhat R. Sun J. (2017a). Forced Degradation of Recombinant Monoclonal Antibodies: A Practical Guide. MAbs 9, 1217–1230. 10.1080/19420862.2017.1368602 28853987 PMC5680805

[B23] Nowak C. Ponniah G. Neill A. Liu H. (2017b). Characterization of Succinimide Stability during Trypsin Digestion for LC-MS Analysis. Anal. Biochem. 526, 1–8. 10.1016/j.ab.2017.03.005 28274724

[B24] Ono M. Horita S. Sato Y. Nomura Y. Iwata S. Nomura N. (2018). Structural Basis for Tumor Necrosis Factor Blockade with the Therapeutic Antibody Golimumab. Protein Sci. 27, 1038–1046. 10.1002/pro.3407 29575262 PMC5980524

[B25] Ouellette D. Chumsae C. Clabbers A. Radziejewski C. Correia I. (2013). Comparison of the *In Vitro* and *In Vivo* Stability of a Succinimide Intermediate Observed on a Therapeutic IgG1 Molecule. mAbs 5, 432–444. 10.4161/mabs.24458 23608772 PMC4169036

[B26] Pace A. L. Wong R. L. Zhang Y. T. Kao Y.-H. Wang Y. J. (2013). Asparagine Deamidation Dependence on Buffer Type, pH, and Temperature. J. Pharm. Sci. 102, 1712–1723. 10.1002/jps.23529 23568760

[B27] Parekh B. S. Berger E. Sibley S. Cahya S. Xiao L. Lacerte M. A. (2012). Development and Validation of an Antibody-dependent Cell-Mediated Cytotoxicity-Reporter Gene Assay. MAbs 4, 310–318. 10.4161/mabs.19873 22531445 PMC3355484

[B28] Reichert J. M. (2012). Marketed Therapeutic Antibodies Compendium. MAbs 4, 413–415. 10.4161/mabs.19931 22531442 PMC3355480

[B29] Rogstad S. Yan H. Wang X. Powers D. Brorson K. Damdinsuren B. (2019). Multi-Attribute Method for Quality Control of Therapeutic Proteins. Anal. Chem. 91, 14170–14177. 10.1021/acs.analchem.9b03808 31618017

[B30] Lu R.-M. Hwang Y.-C. Liu I.-J. Lee C.-C. Tsai H.-Z. Li H.-J. (2020). Development of Therapeutic Antibodies for the Treatment of Diseases. J. Biomed. Sci. 27, 1. 10.1186/s12929-019-0592-z 31894001 PMC6939334

[B31] Scotchler J. W. Robinson A. B. (1974). Deamidation of Glutaminyl Residues: Dependence on pH, Temperature, and Ionic Strength. Anal. Biochem. 59, 319–322. 10.1016/0003-2697(74)90040-2 4407737

[B32] Shanmugaraj B. Siriwattananon K. Wangkanont K. Phoolcharoen W. (2020). Perspectives on Monoclonal Antibody Therapy as Potential Therapeutic Intervention for Coronavirus Disease-19 (COVID-19). Asian Pac. J. Allergy Immunol. 38, 10–18. 10.12932/AP-200220-0773 32134278

[B33] Shealy D. J. Cai A. Staquet K. Baker A. Lacy E. R. Johns L. (2010). Characterization of Golimumab, a Human Monoclonal Antibody Specific for Human Tumor Necrosis Factor α. MAbs 2, 428–439. 10.4161/mabs.12304 20519961 PMC3180089

[B34] Tambuyzer E. Vandendriessche B. Austin C. P. Brooks P. J. Larsson K. Miller Needleman K. I. (2020). Therapies for Rare Diseases: Therapeutic Modalities, Progress and Challenges Ahead. Nat. Rev. Drug Discov. 19, 93–111. 10.1038/s41573-019-0049-9 31836861

[B35] Tamizi E. Jouyban A. (2016). Forced Degradation Studies of Biopharmaceuticals: Selection of Stress Conditions. Eur. J. Pharm. Biopharm. 98, 26–46. 10.1016/j.ejpb.2015.10.016 26542454

[B36] Tebbey P. W. Varga A. Naill M. Clewell J. Venema J. (2015). Consistency of Quality Attributes for the Glycosylated Monoclonal Antibody Humira^®^ (Adalimumab). MAbs 7, 805–811. 10.1080/19420862.2015.1073429 26230301 PMC4622832

[B37] Valliere-Douglass J. Jones L. Shpektor D. Kodama P. Wallace A. Balland A. (2008). Separation and Characterization of an IgG2 Antibody Containing a Cyclic Imide in CDR1 of Light Chain by Hydrophobic Interaction Chromatography and Mass Spectrometry. Anal. Chem. 80, 3168–3174. 10.1021/ac702245c 18355059

[B38] Vatsa S. (2022). In Silico prediction of post-translational Modifications in Therapeutic Antibodies. MAbs 14, 2023938. 10.1080/19420862.2021.2023938 35040751 PMC8791605

[B39] Vlasak J. Bussat M. C. Wang S. Wagner-Rousset E. Schaefer M. Klinguer-Hamour C. (2009). Identification and Characterization of Asparagine Deamidation in the Light Chain CDR1 of a Humanized IgG1 Antibody. Anal. Biochem. 392, 145–154. 10.1016/j.ab.2009.05.043 19497295

[B40] Wang L.-h. Li D.-Q. Fu Y. Wang H.-P. Zhang J.-F. Yuan Z.-F. (2007). pFind 2.0: a Software Package for Peptide and Protein Identification via Tandem Mass Spectrometry. Rapid Commun. Mass. Spectrom. 21, 2985–2991. 10.1002/rcm.3173 17702057

[B41] Wang X. Mathieu M. Brezski R. J. (2018). IgG Fc Engineering to Modulate Antibody Effector Functions. Protein Cell 9, 63–73. 10.1007/s13238-017-0473-8 28986820 PMC5777978

[B42] Xu X. Huang Y. Pan H. Molden R. Qiu H. Daly T. J. (2019). Quantitation and Modeling of post-translational Modifications in a Therapeutic Monoclonal Antibody from Single- and Multiple-Dose Monkey Pharmacokinetic Studies Using Mass Spectrometry. PLOS ONE 14, e0223899. 10.1371/journal.pone.0223899 31618250 PMC6795451

[B43] Lu X. Machiesky L. A. De Mel N. Du Q. Xu W. Washabaugh M. (2020). Characterization of IgG1 Fc Deamidation at Asparagine 325 and its Impact on Antibody-dependent Cell-Mediated Cytotoxicity and FcγRIIIa Binding. Sci. Rep. 10, 383. 10.1038/s41598-019-57184-2 31941950 PMC6962426

[B44] Xie L. Zhang E. Xu Y. Gao W. Wang L. Xie M. H. (2020). Demonstrating Analytical Similarity of Trastuzumab Biosimilar HLX02 to Herceptin^®^ with a Panel of Sensitive and Orthogonal Methods Including a Novel FcγRIIIa Affinity Chromatography Technology. BioDrugs 34, 363–379. 10.1007/s40259-020-00407-0 32072477 PMC7211197

[B45] Yan B. Steen S. Hambly D. Valliere-Douglass J. Bos T. V. Smallwood S. (2009). Succinimide Formation at Asn 55 in the Complementarity Determining Region of a Recombinant Monoclonal Antibody IgG1 Heavy Chain. J. Pharm. Sci. 98, 3509–3521. 10.1002/jps.21655 19475547

[B46] Yang H. Zubarev R. A. (2010). Mass Spectrometric Analysis of Asparagine Deamidation and Aspartate Isomerization in Polypeptides. Electrophoresis 31, 1764–1772. 10.1002/elps.201000027 20446295 PMC3104603

[B47] Yang Y. Guo Q. Xia M. Li Y. Peng X. Liu T. (2015). Generation and Characterization of a Target-Selectively Activated Antibody against Epidermal Growth Factor Receptor with Enhanced Anti-tumor Potency. MAbs 7, 440–450. 10.1080/19420862.2015.1008352 25679409 PMC4622528

[B48] Ying Y. Li H. (2022). Recent Progress in the Analysis of Protein Deamidation Using Mass Spectrometry. Methods 200, 42–57. 10.1016/j.ymeth.2020.06.009 32544593

[B49] Xu Y. Wang D. Mason B. Rossomando T. Li N. Liu D. (2019). Structure, Heterogeneity and Developability Assessment of Therapeutic Antibodies. MAbs 11, 239–264. 10.1080/19420862.2018.1553476 30543482 PMC6380400

[B50] Yu X. C. Joe K. Zhang Y. Adriano A. Wang Y. Gazzano-Santoro H. (2011). Accurate Determination of Succinimide Degradation Products Using High Fidelity Trypsin Digestion Peptide Map Analysis. Anal. Chem. 83, 5912–5919. 10.1021/ac200750u 21692515

[B51] Zhang Y. T. Hu J. Pace A. L. Wong R. Wang Y. J. Kao Y.-H. (2014). Characterization of Asparagine 330 Deamidation in an Fc-Fragment of IgG1 Using Cation Exchange Chromatography and Peptide Mapping. J. Chromatogr. B 965, 65–71. 10.1016/j.jchromb.2014.06.018 24999246

