# Supplementary material for: Identification, Efficacy, and Stability Evaluation of Succinimide Modification With a High Abundance in the Framework Region of Golimumab
Source: Front Chem. 2022 Apr 5;10:826923. doi: 10.3389/fchem.2022.826923 (PMC9017650; doi:10.3389/fchem.2022.826923)
Supplement: Supplementary file 1 [file DataSheet1.docx]

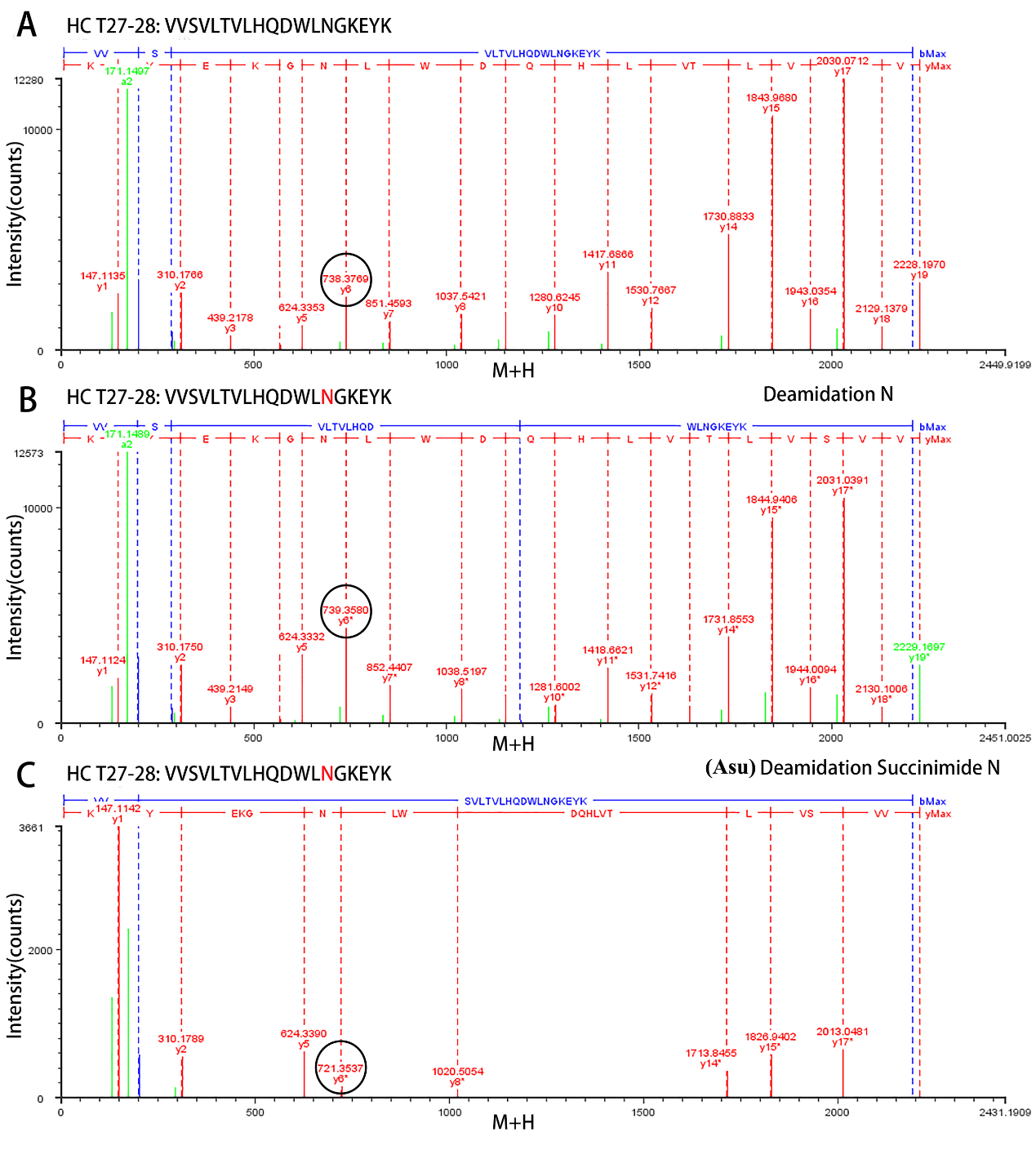


**Supplementary Figure S1. Tandem mass spectra derived by collision-induced dissociation (CID) of the (M + 2H)2+ precursor ions of the peptide HC: H27-28 (VVSVLTVLHQDWLNGKEYK) (A) and its two isoforms: Asu (C), and Asp (B).**

**
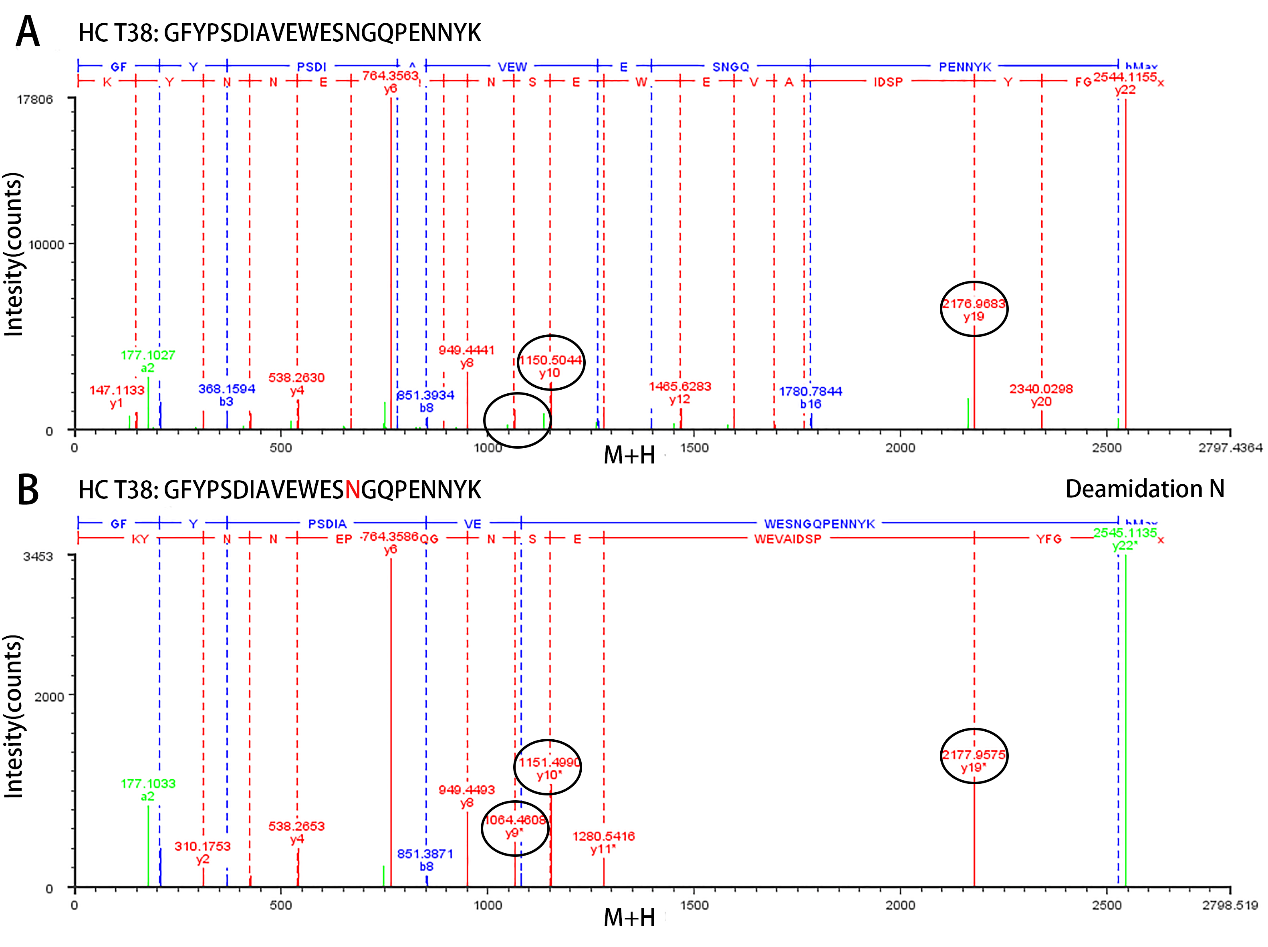
**

**Supplementary Figure S2. Tandem mass spectra derived by CID of the (M + 2H)2+ precursor ions of the peptide HC: H38 (GFYPSDIAVEWESNGQPENNYK) (A) and deamidation (B).**


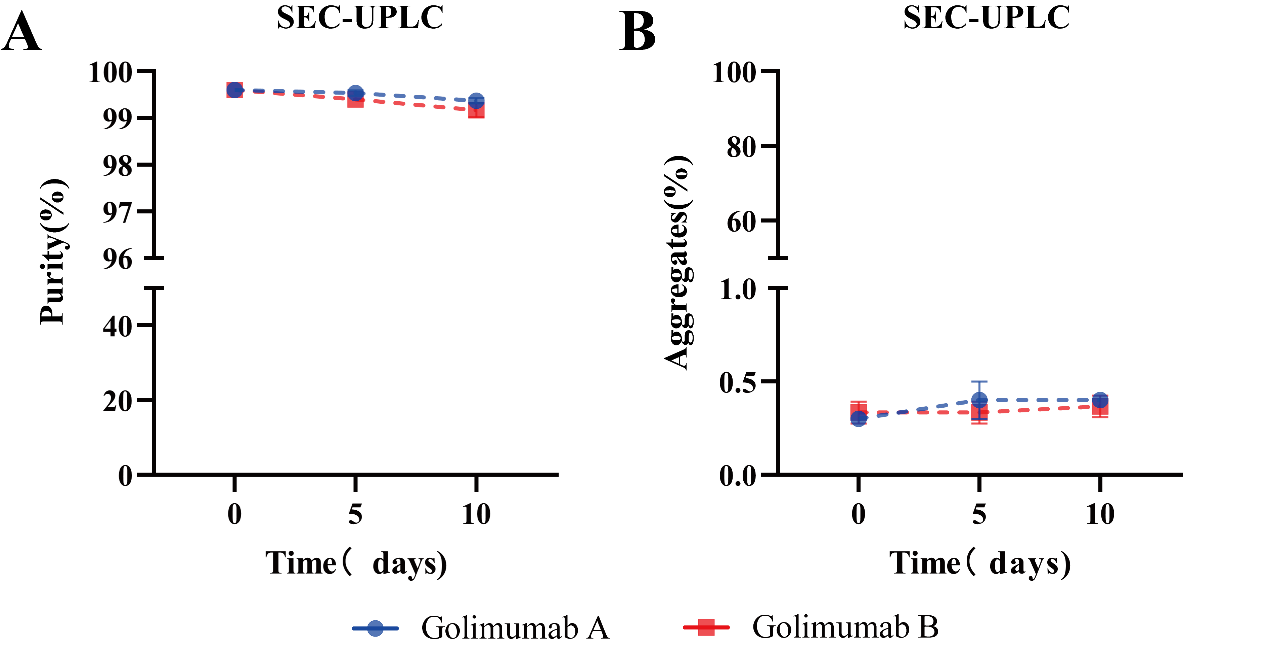


**Supplementary Figure S3. Forced degradation evaluation of the Asu modification with a high abundance in golimumab. The purity (A) and aggregates (B) of Golimumab A and Golimumab B were anlyzed by SEC-UPLC at the (0, 5, 10) day time points, respectively.**
